# MicroRNA-207 enhances radiation-induced apoptosis by directly targeting akt3 in cochlea hair cells

**DOI:** 10.1038/cddis.2014.407

**Published:** 2014-10-02

**Authors:** P-x Tan, S-s Du, C Ren, Q-w Yao, R Zheng, R Li, Y-w Yuan

**Affiliations:** 1 Department of Radiation Oncology, Nanfang Hospital, Southern Medical University, Guangzhou, Guangdong 510515, China

## Abstract

MicroRNAs (miRNAs) have important roles in various types of cellular biological processes. Our study aimed to determine whether miRNAs function in the regulation of ionizing radiation (IR)-induced cell death in auditory cells and to determine how they affect the cellular response to IR. Microarray and qRT-PCR were performed to identify and confirm the differential expression of miRNAs in the cochlea hair cell line HEI-OC1 and *in vivo* after IR. Upregulation or downregulation of miRNAs using miRNA mimics or inhibitor were detected to characterize the biological effects of the indicated miRNAs. Bioinformatic analyses, luciferase reporter assays and mRNA knockdown were performed to identify a miRNA target gene. We determined that miR-207 was significantly upregulated after IR. MiR-207 enhances IR-induced apoptosis and DNA damage in HEI-OC1 cells. Furthermore, Akt3 was confirmed to be a direct target of miR-207. Downregulation of Akt3 mimics the effects of miR-207. MiR-207 enhances IR-induced apoptosis by directly targeting Akt3 and anti-miR-207 may have a potential role in protecting cochlea hair cells from IR.

Radiotherapy (RT) is one of the most important treatments for head and neck (HN) cancers. Although the technologies for RT have greatly improved in recent years, the incidence of side effects induced by RT remains high. Sensorineural hearing loss (SNHL) is considered to be a principal complication of RT for HN and markedly affects the quality of life for patients with HN cancers.^1^ It has been demonstrated that the death of cochlea hair cells is responsible for ionizing radiation (IR)-induced SNHL.^2, 3, 4, 5, 6^ Regulators, such as p53, reactive oxygen species and c-Jun N-terminal kinases are known to have important roles in apoptosis of irradiated hair cells.^7, 8, 9, 10^ *N*-acetylcysteine,^11^ epicatechin^10^ and metformin^12^ have been previously demonstrated to be effective in reducing apoptosis in irradiated hair cells. Because studies have been limited, the mechanisms underlying IR-induced auditory cell death remain unclear and require further investigation.

MicroRNAs (miRNAs) are a class of short noncoding RNAs. Some miRNAs have been extensively investigated. These RNAs repress translation or the stability of target mRNAs by binding to the 3'-untranslated regions (UTRs) of the mRNAs with imperfect complementarity.^13,14^ By regulating gene expression at the post-transcriptional level, miRNAs have important roles in various types of cellular biological processes, including responses to IR. However, most published studies on miRNAs regulating radiosensitivity have examined cancer cells, and little attention has been devoted to normal cells. Moreover, the relationship between miRNAs and IR-induced cochlea hair cell death has not been investigated to date.

In this study, we aimed to investigate IR-responsive miRNAs by analyzing the miRNA expression profile in the auditory cell line HEI-OC1 and identified miR-207 as an IR-inducible miRNA. MiR-207 enhanced apoptosis by increasing DNA damage in irradiated HEI-OC1 cells. Further investigation revealed that miR-207 negatively regulated Akt3 as a direct target. Thus, we provide a new mechanism for IR-induced apoptosis in cochlea hair cells. Taken together, our findings may help to develop a potential protectant for IR-induced SNHL.

## Results

### MiR-207 expression is induced by IR and inhibits cell growth

**Figure 1:**
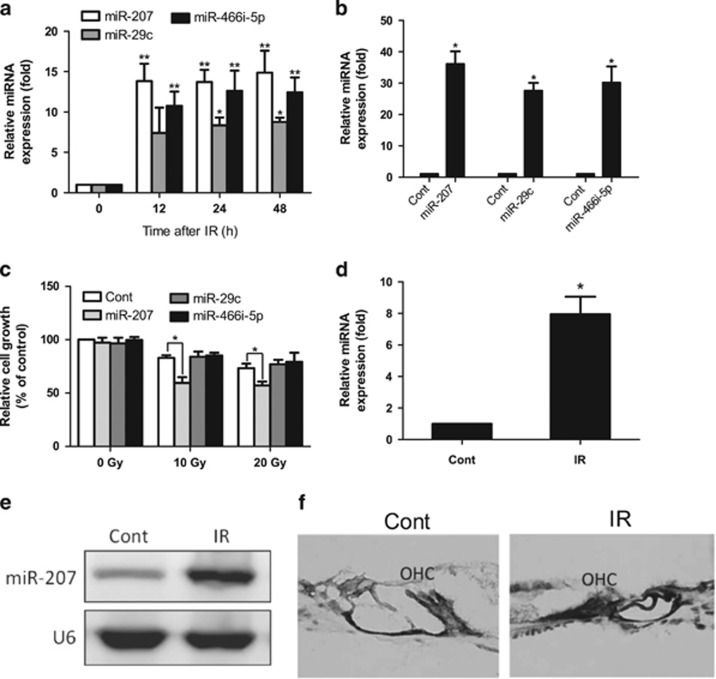
miR-207 expression is induced by IR and inhibits cell growth. (**a**) qRT-PCR was performed to confirm the upregulated expression of miR-207, miR-29c and miR-466i-5p in HEI-OC1 cells at 12, 24 and 48 h after 20 Gy irradiation. U6 spliceosomal RNA was used for normalization. Error bar, S.D.; *P<0.05 (*n*=3) *versus* the nonirradiated (0 h) group, **P<0.001 (*n*=3) *versus* the nonirradiated (0 h) group. (**b**) HEI-OC1 cells were transfected with miR-207, miR-29c and miR-466i-5p independently. qRT-PCR was performed to detect the level of the indicated miRNAs at 48 h after transfection. Error bar, S.D.; *P<0.01 (*n*=3) *versus* control. (**c**) HEI-OC1 cells transfected with miR-207, miR-29c, miR-466i-5p and control miRNAs were subjected to the MTT assay at 24 h after IR (10, 20 Gy) or without IR. Error bar, S.D.; *P<0.001 (*n*=3) *versus* control. (**d**) qRT-PCR, (**e**) northern blotting and (**f**) *in situ* hybridization were performed to confirm the upregulated expression of miR-207 in cochlea at 24 h after 20 Gy irradiation on mice. U6 was used for normalization. Error bar, S.D.; *P<0.001 (*n*=5) *versus* control. OHC= outer hair cell

A differential miRNA expression profile between irradiated and nonirradiated HEI-OC1 cells was determined using microarray. These results revealed that three miRNAs were upregulated, and nine miRNAs were downregulated after IR when taking into account a fold-change>6.3 (*lg*0.8) and *P*<0.05 (Table 1). We focused on the three upregulated miRNAs, including miR-207, miR-29c and miR-466i-5p, for further investigation. The qRT-PCR results confirmed that all these miRNAs were significantly upregulated at 12, 24 and 48 h after IR and the high expression remained stable (Figure 1a). After we successfully transfected miR-207, miR-29c and miR-466i-5p mimics individually into HEI-OC1 cells (Figure 1b), an methyl thiazolyl tetrazolium (MTT) assay was performed. Upregulation of miR-207 significantly inhibited cell growth in cells after IR (10, 20 Gy) but not in cells without IR, whereas miR-29c or miR-466i-5p overexpression did not exhibit any growth effect in irradiated or nonirradiated cells (Figure 1c). To confirm the expression of miR-*207 in vivo*, we performed assays on irradiated and control cochleas. Results from qRT-PCR, northern blotting and *in situ* hybridization (ISH) were identical and further verified the upregulation of miR-207 in irradiated cochleas (Figures 1d–f). On the basis of this finding, further studies were performed to determine how miR-207 affects cell growth.

### MiR-207 enhances IR-induced apoptosis

**Figure 2:**
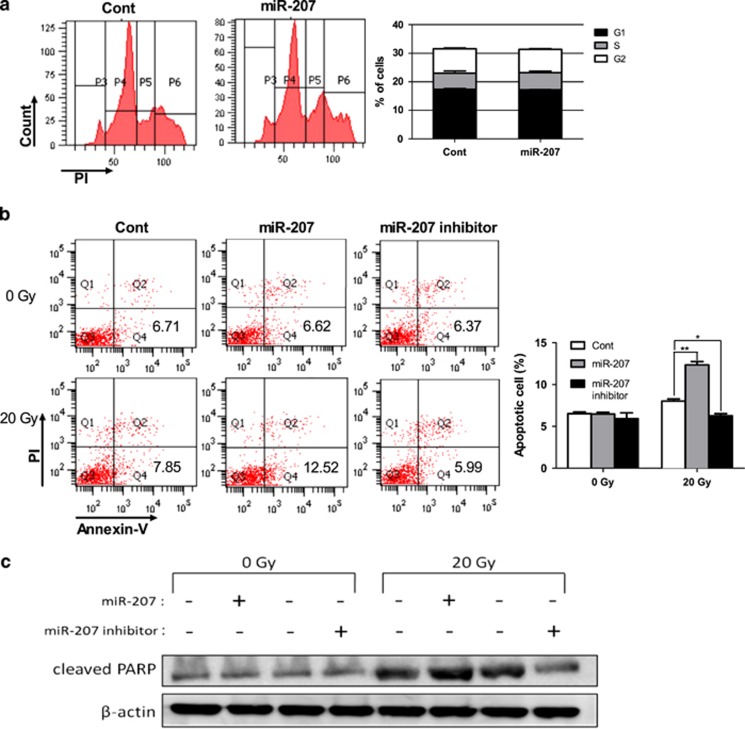
miR-207 enhances IR-induced apoptosis. HEI-OC1 cells were transfected with miR-207, miR-207 inhibitor or control miRNAs prior to subsequent experiments. (**a**) Cell cycle analysis was performed 24 h after IR (20 Gy) to examine the effect of miR-207 on cell cycle distribution. (**b**) The apoptosis assay using flow cytometry was performed 24 h after transfected cells were treated with 0 Gy IR or without IR. Error bars, S.D.; *P=0.001 (*n*=3) *versus* the control group, **P<0.001 (*n*=3) *versus* control. (**c**) The expression levels of cleaved PARP were detected by western blotting analyses using an anti-cleaved PARP antibody in transfected cells at 24 h after 20 Gy IR or without IR

The flow cytometry results for cell cycle analysis showed that populations of G1, S and G2 phases were not significantly different between miR-207 transfected and control cells after IR (Figure 2a), which indicated that miR-207 did not affect the distribution of cell cycle in irradiated cells. Next, we investigated whether miR-207 affected apoptosis in HEI-OC1 cells. The flow cytometry results for apoptosis indicated an upregulation of miR-207 significantly enhanced apoptosis compared with control in irradiated cells, whereas inhibition of miR-207 significantly mitigated apoptosis (Figure 2b). In cells without IR, no differences were found between groups treated with miR-207, miR-207 inhibitor or control. To confirm the apoptosis-enhancement effect of miR-207, western blotting analyses were performed. MiR-207 moderately increased the expression of cleaved PARP after IR, whereas inhibition of miR-207 greatly repressed cleaved PARP expression (Figure 2c). Furthermore, in cells treated without IR, the level of miR-207 did not affect the expression of cleaved PARP. On the basis of these studies, we concluded that miR-207 enhanced apoptosis, which only occurred in cells with IR.

### MiR-207 enhances IR-induced DNA damage

**Figure 3:**
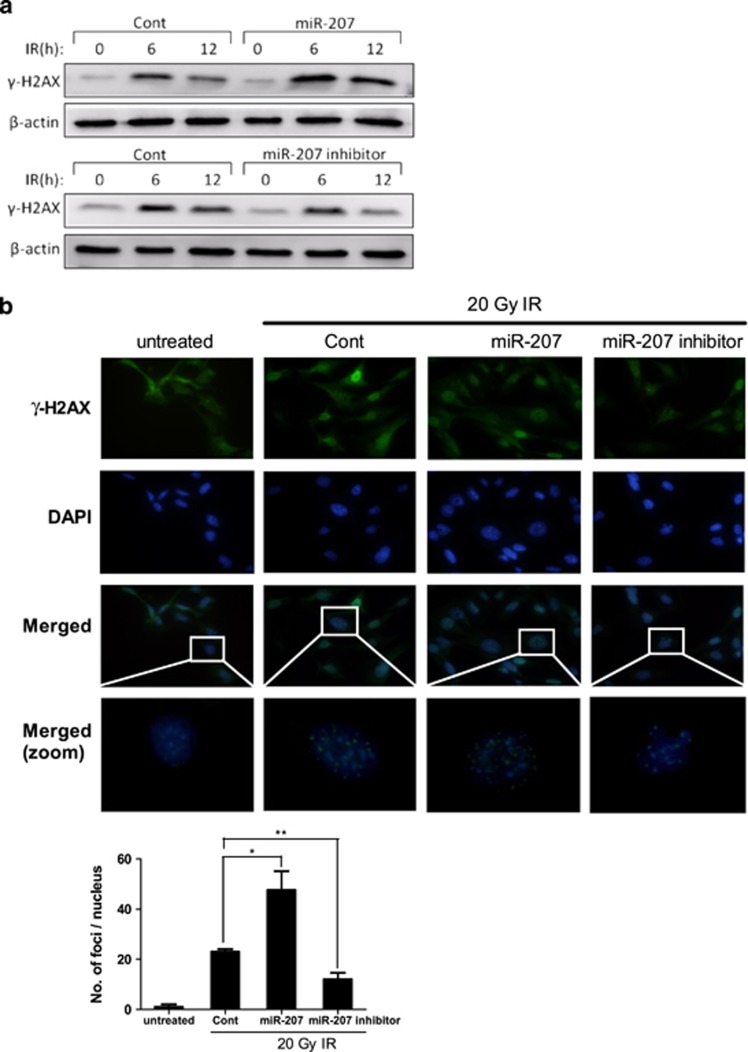
miR-207 enhances IR-induced DNA damage. HEI-OC1 cells were transfected with miR-207, miR-207 inhibitor or control miRNAs prior to subsequent experiments. (**a**) Transfected cells were treated with 20 Gy IR or without IR, and collected at 6 and 12 h after IR. Expression levels of *γ*-H2AX (green) were detected by western blotting analyses using an anti-*γ*-H2AX antibody. (**b**) Immunofluorescent staining for *γ*-H2AX was performed at 12 h after 20 Gy IR of transfected cells. Error bar, S.D.; *P=0.001 (*n*=3) *versus* control; **P<0.05 (*n*=3) *versus* control

Next, we investigated whether increased apoptosis by miR-207 is associated with an enhancement in DNA damage. Transfection with miR-207 resulted in higher *γ*-H2AX expression at 6 and 12 h after IR compared with control. In contrast, transfection with miR-207 inhibitors resulted in significantly lower *γ*-H2AX expression independent of time (6 or 12 h) after IR (Figure 3a). We also assessed the DNA damage by quantifying *γ*-H2AX foci after staining. HEI-OC1 cells with upregulated miR-207 expression showed significantly more foci in the nucleus, whereas cells with downregulated miR-207 expression showed the opposite result. Taken together, these results suggest that miR-207 significantly enhanced IR-induced DNA damage.

### Akt3 is a direct target of miR-207

**Figure 4:**
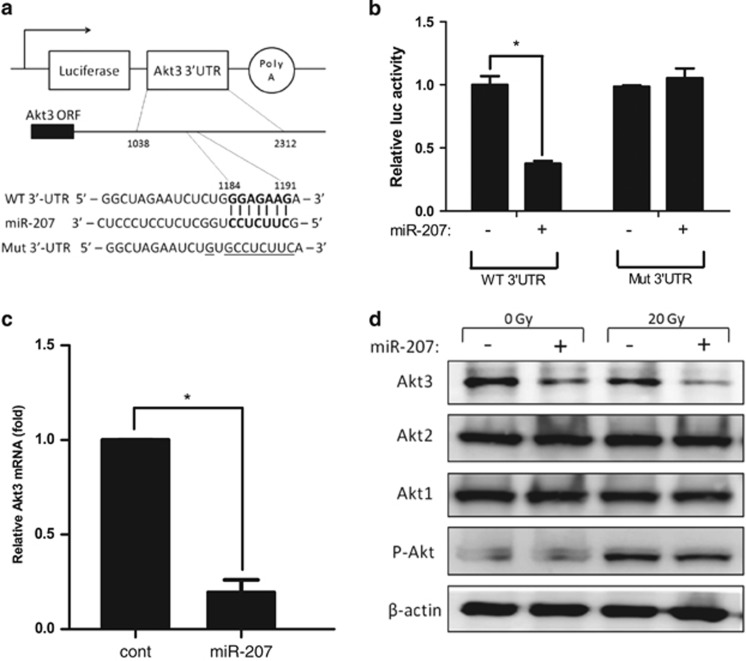
Akt3 is a direct target of miR-207. (**a**) The structure of the reporter plasmid, nucleotide of Akt3 3'-UTR complementary to the seed sequence of miR-207 and the mutant sequence. (**b**) The relative luciferase activity in HEI-OC1 cells was determined after co-transfection with Akt3 3'-UTR or Mut-Akt3 3'-UTR plasmids and miR-207 or control. For each sample, the relative luciferase activity was normalized with firefly luciferase activity. Error bars, S.D.; *P=0.001 (*n*=3) *versus* control. (**c**) Akt3 mRNA expression was determined using qRT-PCR in cells transfected with miR-207 or control miRNAs. Error bars, S.D.; *P<0.01 (*n*=3) *versus* control. (**d**) Expression levels of Akt3 and other related proteins were determined by western blotting analyses using indicated antibodies in miR-207 or control miRNA-transfected cells. The transfected cells were collected at 24 h after 20 Gy IR or without IR

To identify the target mRNA of miR-207, we used five miRNA target prediction programs,^15^ including EIMMo, miRanda, miTarget, PicTar and TargetScan. As shown in Figure 4a, the complementary sequence for the seed region of miR-207 was at position 1184-1191 of Akt3 3'-UTR. A reporter (WT 3'-UTR) containing the exact complementary sequence in the Akt3 3'-UTR fragment and another reporter (Mut 3'-UTR) containing mutated nucleotides of the complementary sequence were constructed (Figure 4a). Expectedly, co-transfection with miR-207 and WT 3'-UTR reporter significantly reduced luciferase activity compared with control (Figure 4b). In addition, Akt3 mRNA and protein levels significantly decreased after miR-207 transfection (Figures 4c and d). However, the expression of other Akt isoforms, including Akt1 and Akt2, remained unchanged (Figure 4d). Moreover, P-Akt increased markedly after IR, but decreased moderately in cells transfected with miR-207. Taken together, these results revealed that Akt3 is a direct target of miR-207.

### Downregulation of Akt3 mimics the effects of miR-207

**Figure 5:**
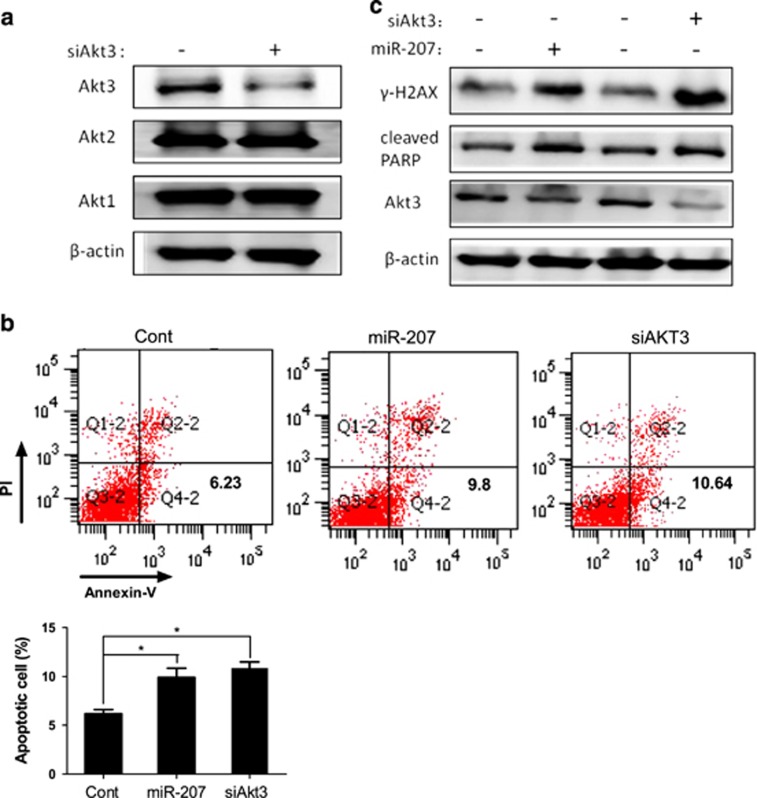
Downregulation of Akt3 mimics the effects of miR-207. (**a**) Expression levels of Akt3 and other Akt isoforms, such as Akt1 and Akt2, were determined using western blotting analyses in HEI-OC1 cells transfected with siAkt3 or control. (**b**) HEI-OC1 cells were transfected with miR-207, siAKT3 or control. The apoptosis assay using flow cytometry was performed 24 h after transfected cells were treated with 20 Gy IR. Error bars, S.D.; *P≤0.001 (*n*=3) *versus* control. (**c**) Expression of *γ*-H2AX, cleaved PARP and Akt3 proteins were determined by western blotting analyses using the indicated antibodies in transfected cells after 20 Gy IR. The cells used to determine the expression level of *γ*-H2AX and cleaved PARP and Akt3 were collected at 12 h and 24 h after IR, respectively

We next examined whether downregulation of Akt3 exhibited similar effects of miR-207. First, we specifically repressed the expression of Akt3 protein, but not Akt1 or Akt2 (Figure 5a). Apoptosis analysis showed that HEI-OC1 cells transfected with miR-207 or siAkt3 exhibited significantly greater apoptosis compared with control (Figure 5b). At the protein level, western blotting analyses detected the expression of *γ*-H2AX as well as cleaved PARP, which increased similarly between cells transfected with miR-207 and siAkt3 (Figure 5c).

## Discussion

MiRNAs are indispensible for cochlea hair cell maintenance and survival.^16^ The expression of miRNA changes while the cochlea is under various stress, eventually causing cell death and SNHL.^17^ Oxidative stress upregulates 35 miRNAs and downregulates 40 miRNAs in HEI-OC1 cells.^18^ The miR-183/*Taok1* target pair and miR-34 family are found implicated in cochlear responses to acoustic trauma and kanamycin ototoxicity, respectively.^19,20^ In our study, miR-207, miR-29c and miR-466i-5p were identified as upregulated miRNAs in HEI-OC1 cells after IR, and miR-207 was confirmed to be the only one that affects cell viability. These evidences show that different stress may cause different miRNA expression in cochlea cells, which is probably because different miRNAs take part in different cellular processes. To the best of our knowledge, miR-207 has not been thoroughly investigated. MiR-207 was found to be downregulated in liver tissue after partial hepatectomy in mice^21^ and upregulated in a neuronal cell line (MN9D) with 6-hydroxydopamine (6-OHDA) treatment, a component of a neurotoxin.^22^ Although these studies demonstrated changes in miR-207 expression, they did not investigate the function of miR-207. Our study reveals the biological function of miR-207 and proposes a miRNA correlated to IR-induced injury in auditory cells.

Further studies have revealed that inhibition of cell growth by miR-207 is caused by increased cell apoptosis rather than cell cycle arrest. Moreover, an enhancement of apoptosis by miR-207 was only observed in irradiated cells, which suggested that this change is associated with IR-induced DSBs. It is known that DNA is the major target of radiation effects. The unsuccessful repair of DSBs may result in lethal consequences, such as apoptosis, for cells.^23^ Moreover, *γ*-H2AX is one of the earliest markers of DSBs after IR.^24^ In the present study, the expression of *γ*-H2AX was greatly increased and was sustained in cells upregulated with miR-207. This finding indicated that miR-207 enhanced IR-induced DNA damage, which results in enhanced apoptosis.

Akt3 has been shown to be a direct target of miR-207 in our study. Isoforms of the Akt family, including Akt3, Akt1 and Akt2, share a high degree of structural similarity, but express and function differently in specific cell types and biological processes. Akt3 has been studied extensively in cancer cells for its effects on cancer development, proliferation and migration;^25,26^ however, little is known regarding its function in normal cells. In our study, Akt3 was found to be specifically inhibited by miR-207. Inhibition by siAkt3 resulted in an enhancement of apoptosis and DSBs in irradiated HEI-OC1 cells, similar to results obtained with miR-207 treatment. This indicated Akt3 potentially has an important role in miR-207-mediated IR response in auditory cells. In addition, Akt1 and Akt2 were shown to be important regulators in the activation of DNA-dependent protein kinase, a key enzyme of nonhomologous end joining of the DNA repair pathway.^27, 28, 29^ Akt3 may share a DSB repair pathway with Akt1 and Akt2, and further studies are required.

As previously mentioned, the therapeutic options against IR-induced cochlea hair cell death are notably limited. Thus, the identification of new therapeutic agents is important. Although we have identified many genes related to apoptosis induced by IR, it is not easy to directly manipulate these genes. Among the regulators of protein-coding genes, miRNA is an ideal choice. In our study, specific inhibition of miR-207 exhibits the exciting potential to protect HEI-OC1 cells from IR by reducing apoptosis and DSBs. Thus, further studies are required to confirm the protective effect of anti-miR-*207 in vivo*.

## Conclusions

In summary, our study is the first to indicate that overexpression of miR-207 enhances apoptosis in irradiated auditory cells. Moreover, the enhancement of apoptosis is potentially caused by the disruption in DSBs repair. We also determined that specific inhibition of miR-207 significantly mitigated damages induced by IR on HEI-OC1 cells, which may represent a novel strategy in protecting IR-induced SNHL. Furthermore, we confirmed Akt3 is a direct target for miR-207. Thus, additional studies are needed to clearly reveal the molecular mechanisms underlying the role of Akt3 in miR-207-enhanced apoptosis after IR.

## Materials and Methods

### Cell culture

The HEI-OC1 cell line was generously provided by F. Kalinec (House Ear Institute, Los Angeles, CA, USA). It is a conditionally immortalized organ of the Corti-derived epithelial cell line,^30^ which has been used to investigate the cellular and molecular mechanisms of ototoxicity induced by drugs, noise or irradiation. This cell line was maintained in high-glucose Dulbecco's modified Eagle's medium (Gibco, Cergy-Pontoise, France) containing 10% fetal bovine serum (Gibco) without antibiotics at 33 °C under 10% CO_2_ in an incubator.

### Animals

C57BL/6 mice were purchased from the Southern Medical University Laboratory. Animal care and killing were conducted according to methods approved by the Southern Medical University Animal Care and Use Committee, following guidelines of National Institute of Health for use of laboratory animals. 10 C57BL/6 mice were divided into two groups (control group and irradiation group).

### Irradiation

The HEI-OC1 cells were irradiated at a distance of 100 cm from the source to the axis using a 6-MV linear accelerator (LINAC; 2300EX; Varian Co., Palo Alto, CA, USA) at a dose rate of 5.0 Gy/min. The mice of radiation group were placed and fixed in the prone position on a plate after they were anaesthetized with 40 mg/kg 0.1% pentobarbital sodium. The mice then received irradiation restricted to the head. A single dose of 20 Gy was delivered by opposed beams bilaterally with a distance of 100 cm from the source to the axis.

### miRNA microarray

The expression of miRNAs in IR-treated versus untreated cells was analyzed using miRNA microarray. The total RNA was extracted from the untreated cells or cells 24 h post IR (20 Gy) using Trizol (Invitrogen, Carlsbad, CA, USA) according to the instructions provided by the manufacturer. Determination of the quantity and quality of the extracted RNA and the microarray experiment were performed by Guangzhou RiboBio Co. Ltd. using Mouse & Rat miRNA OneArray V3.0 (Phalanx Biotech Company, Hsinchu, Taiwan).

### qRT-PCR

After isolation from the IR-treated or untreated cells or cochlea, the total RNA was reverse-transcribed into cDNA using the PrimeScript RT reagent kit (TaKaRa, Dalian, China) according to the manufacturer's instructions. qPCR was performed using the ABI PRISM 7500 Fast Real–Time PCR System (Perkin Elmer/Applied Biosystems, Rotkreuz, Switzerland) with a SYBR Premix Ex Taq II kit (TaKaRa). The small noncoding RNA U6 and housekeeping gene GADPH were used as an internal control for miRNA and Akt3 quantification, respectively. The sequences of the gene-specific primers used for qPCR are shown as follows: miR-207: forward 5′-ACACTCCAGCTGGGGCTTCTCCTGGCTCTCC-3′ miR-29c: forward 5′-ACACTCCAGCTGGGTAGCACCATTTGAAAT-3′ miR-466i-5p: forward 5′-ACACTCCAGCTGGGTGTGTGTGTGTGTG-3′ universal reverse primer for miRNAs: 5′-TGGTGTCGTGGAGTCG-3′ Akt3: forward 5′-ACCGCACACGTTTCTATGGT-3′, reverse 5′-CCCTCCACCAAGGCGTTTAT-3′ U6: forward 5′-CTCGCTTCGGCAGCACA-3′, reverse 5′-AACGCTTCACGAATTTGCGT-3′ GADPH: forward 5′-GGATTTGGTCGTATTGGG-3′, reverse 5′-GTGGCTGGGGCTCTACTTC-3′. All reactions were performed in triplicate for each sample.

### Northern blotting

The locked nucleic acid (LNA) probe for miR-207 labeled with digoxigenin (DIG Oligonucleotide 3'-End Labeling Kit; Roche, Stockholm, Sweden) were purchased from Exiqon Co (Vedbaek, Denmark). U6 RNA was detected using a DIG-labeled U6 DNA probe. Northern blotting was performed as described.^31^ Total RNA from cochleas was resolved by 15% denaturing polyacrylamide gel electrophoresis and transferred by electroblotting to membranes. Blots were pre-hybridized and hybridized by overnight incubation in buffer containing 0.2 nM DIG-labeled probes at 52 °C for miR-207 LNA probe or 25 °C for the U6 DNA probe. Blots were stringently washed twice in SDS for 30 min at 52 °C for miR-207 LNA probe or 25 °C for the U6 DNA probe, rinsed in wash buffer, and incubated in block buffer for 30 min. Subsequently, blots were incubated with anti-DIG-AP Fab fragment in block buffer for 1 h, washed in wash buffer and detection buffer separately. Anti-DIG-AP was detected using CDP-star chemiluminescent substrate for alkaline phosphatase (AP). Blots were stripped by incubation at 70 °C in 0.1 × SSC containing 1% SDS and probed up to three times.

### Parafin section ISH

ISH was performed to confirm the expression of miR-207 in cochlea hair cells *in vivo*. Cochleas of C57BL/6 mice were carefully and quickly resected, followed by removing the stapes from the oval window and opening the cochlea apex. We then placed the cochleas in fresh 4% paraformaldehyde (PFA) in PBS overnight at 4 °C. After decalcification with 0.1 M ethylenediaminetetraacetic for 3 days at 4 °C, the cochleas were embedded in paraffin and sectioned at 5 *μ*m. ISH was performed as described.^32^ Briefly, sections were hybridized with labeled LNA probe, which was detected using AP-conjugated sheep anti-DIG Fab fragment and BM Purple AP Substrate (Roche). The sections were then fixed by 4% PFA for 20 min finish with milliQ water (Millipore, Billerica, MA, USA) rinses. Slides were coverslipped with Aquamount (Merck, Darmstadt, Germany). The slides were observed using a light microscope.

### Transfection

MiR-207 mimics, miR-207 inhibitor, miR-29c mimics, miR-466i-5p mimics, siAkt3 and control were purchased from RiboBio Co. After the cells were seeded in six-well plates, transfection was performed using Lipofectamine 2000 (Invitrogen) according to the instructions provided by the manufacturer. Experiments performed on transfected cells were carried out 48 h post transfection.

### MTT assay

This assay was performed to examine the viability of HEI-OC1 cells. The transfected cells were seeded in 96-well plates at a density of 1 × 10^4^ cells/well followed by radiation treatment. Next, 20 *μ*l of 5 mg/ml MTT (Sigma, St. Louis, MO, USA) was added to each well at 48 h post IR (10, 20 Gy) or without IR treatment. After culturing for 4 h, the medium was replaced with 150 *μ*l/well dimethylsulfoxide (Sigma) and vortexed for 10 min. The optical density value was measured at 490 nm and the wells that did not contain cells that were used as blanks. Each experiment was performed in triplicate.

### Immunofluorescent staining for *γ*-H2AX

We estimated the DNA damage by performing immunofluorescent staining for *γ*-H2AX. MiR-207-mimics-transfected, miR-207-inhibitor-transfected and control-transfected cells were grown on polylysine-coated coverglasses. At 12 h after radiation (20 Gy), the cells were fixed in 4% PFA for 30 min, permeabilized in 0.25% triton X-100 and blocked for 30 min in 1% goat serum. The cells were then incubated overnight at 4 °C with anti-*γ*H2AX primary antibody (1 : 100 dilution; Abcam, San Francisco, CA, USA). Next, the cells were washed in PBS, and rabbit anti-mouse AlexaFlour-488 secondary antibody (1 : 200 dilution; Abcam) was applied for 1 h at room temperature in the dark. After three 5-min washes in PBS, the samples were mounted in fluorescence mounting medium with DAPI. The cells were observed using a fluorescence microscope (Olympus, Shinjuku-ku, Tokyo, Japan), and the *γ*-H2AX foci were manually quantified in at least three individual fields of ~100 cells.

### Cell cycle analysis using flow cytometry

The transfected cells were collected at 24 h after IR (20 Gy) or without IR. Next, cells were washed with PBS once and resuspended in PBS containing 0.2% Triton X-100 for permeabilization and propidium iodide (PI) staining. After incubation for 30 min, the cells were analyzed using flow cytometry. Each experiment was performed in triplicate.

### Apoptosis analysis using flow cytometry

The transfected cells were collected at 24 h post IR (20 Gy) or without IR treatment. Cells undergoing apoptosis were determined by staining with Alexa Fluor 488 annexin V and PI according to the protocol provided by the manufacturer (Invitrogen). Each experiment was performed in triplicate.

### Western blotting

Cells were collected and western blot analyses were performed as previously described.^33^ *β*-actin was used as the loading control. The primary antibodies used for western blotting included: rabbit anti-cleaved PARP (#9544, Cell Signaling, Beverly, MA, USA), anti-H2AX (#9718, Cell Signaling), anti-Akt1 (#9514, SBA), anti-Akt2 (#8715, SBA), anti-Akt3 (#4059, Cell Signaling), anti-pAkt (#4060, Cell Signaling) and anti-β-actin (#4970, Cell Signaling).

### Bioinformatic analyses

We obtained the mature sequence of miR-207 from the miRNA database (http://www.mirbase.org/). EIMMo (http://www.mirz.unibas.ch/ElMMo2/), miRanda (http://www.microrna.org), miTarget (http://cbit.snu.ac.kr/~miTarget/), PicTar (http://pictar.mdc-berlin.de/) and TargetScan (http://www.targetscan.org/) was used to predict the target gene of miR-207.

### Reporter plasmid constructs and luciferase reporter assays

We confirmed the direct target of miR-207 by performing this assay. The 3′-UTR of the Akt3 gene containing the predicted target sites of miR-207 was amplified by PCR using the primers 5′-TCACAGATCATTGCCTGCGT-3′ for Akt3-3'-UTR-forward and 5′-TCCCACACCTCGGTTCTACT-3′ for Akt3-UTR-reverse was cloned into the Renilla luciferase gene (pLUC-REPORT vector, Promega, Madison, WI, USA). A mutant 3'-UTR of Akt3 with a mutated sequence (5′-…AAUCUGUGCCUCUUCAGAUGCUGUGA…-3′, the mutated sites are underlined) in the complementary site for the miR-207 seed region was also amplified and cloned into the pLUC-REPORT vector. For the reporter luciferase assay, HEI-OC1 calls were cultured in 96-well plates and co-transfected with pLUC-3'UTR-Akt3 (Akt3-WT) or pLUC-3'UTR-Mut-Akt3 (Akt3-Mut) and miR-207 mimics or control with Lipofectamine 2000 (Life Technologies, Carlsbad, CA, USA). Forty-eight hours after transfection, cells were assayed for luciferase activity with the Dual-Luciferase Assay System (Promega) according to the manufacturer's instructions. Independent assays were performed in triplicate.

### Statistical analyses

Data were analyzed using the Student's *t*-test or one-way ANOVA for statistical significance. Statistical evaluations are presented as the mean±S.D., and a *P-*value of <0.05 was considered to be statistically significant.

## Figures and Tables

**Table 1:** Differential miRNAs expression in HEI-OC1 cells after irradiation

	**miRNA ID**	**lg (ratio)**	***P*-value**
Upregulated	mmu-miR-207	1.13	3.73E−05
	mmu-miR-29c	0.96	4.96E−03
	mmu-miR-466i-5p	3.00	4.51 E−05
Downregulated	mmu-miR-101a-5p	−1.04	1.03E−05
	mmu-miR-1247-5p	−1.19	2.12E−05
	mmu-miR-1899	−1.12	4.99E−06
	mmu-miR-222	−1.22	1.80E−04
	mmu-miR-3473d	−1.37	6.99E−05
	mmu-miR-375-5p	−1.24	8.82E−06
	mmu-miR-491-5p	−1.22	1.74 E−04
	mmu-miR-5100	−1.09	7.67 E−04
	mmu-miR-719	−1.11	2.41 E−04

## Abbreviations

IR: ionizing radiation
RT: radiotherapy
HN: head and neck
SNHL: sensorineural hearing loss
UTRs: untranslated regions
MTT: methyl thiazolyl tetrazolium
ISH: *in situ* hybridization
